# *Gavialis* from the Pleistocene of Thailand and Its Relevance for Drainage Connections from India to Java

**DOI:** 10.1371/journal.pone.0044541

**Published:** 2012-09-18

**Authors:** Jeremy E. Martin, Eric Buffetaut, Wilailuck Naksri, Komsorn Lauprasert, Julien Claude

**Affiliations:** 1 School of Earth Sciences, University of Bristol, Bristol, United Kingdom; 2 Palaeontological Research and Education Centre, Mahasarakham University, Maha Sarakham, Thailand; 3 Laboratoire de Géologie de l'Ecole Normale Supérieure, Centre National de la Recherche Scientifique, Unité Mixte de Recherche 8538, Paris, France; 4 Department of Biology, Faculty of Science, Mahasarakham University, Maha Sarakham, Thailand; 5 Institut des Sciences de l'Evolution de Montpellier, Centre National de la Recherche Scientifique, Institut de Recherche pour le Développement, Université de Montpellier 2, Montpellier, France; Ludwig-Maximilians-Universität München, Germany

## Abstract

**Background:**

The genus *Gavialis* comprises a single living but endangered species, *G. gangeticus*, as well as fossil species recorded in the Miocene to Pleistocene deposits of the Indian subcontinent. The genus is also represented in the Pleistocene deposits of Java by the species *G. bengawanicus*, which was recently recognized to be valid. Surprisingly, no detailed report of the genus exists between these two provinces and the recent evolutionary history of *Gavialis* is not understood.

**Methodology/Principal Findings:**

We report new material consisting of skull and mandibular remains of *Gavialis* from the Early Pleistocene of Khok Sung, Nakhon Ratchasima Province, northeastern Thailand. The *Gavialis* material described herein is attributed to *Gavialis* cf. *bengawanicus* and sheds new light on the occurrence of the genus in mainland SE Asia.

**Conclusions/Significance:**

Comparison of this new material with other species referred to the genus *Gavialis* led us to preliminary restrict the content of the genus to three species, namely *G. gangeticus* Gmelin, *G. bengawanicus* Dubois and *G. lewisi* Lull. The occurrence of *G.* cf. *bengawanicus* in Thailand allows us to propose a scenario for the dispersal of *Gavialis* from Indo-Pakistan to Indonesia, thus bridging a geographical gap between these two provinces. Dispersal by sea appears a less likely possibility than dispersal through fluvial drainages.

## Introduction

Studies on Neogene vertebrates from SE Asia have mostly concerned mammal faunas (e.g. [1]–[3]). Although fossil remains of turtles and crocodilians were reported from the Neogene of Burma (now Myanmar) as early as the 1820s [4], [5], the herpetofaunas of this period have received very little attention, with a few contributions dealing with turtles [6]–[12] and virtually no paper describing crocodilian remains, with the exception of *Gavialis bengawanicus* and *Crocodylus ossifragus* from the Pleistocene of Java [13]–[15]. Recently, fragmentary remains of *Gavialis* sp. have been reported from a nearby locality at Tha Chang sandpit in Nakhon Ratchasima Province [12]. These pits have not been precisely dated but yielded faunas reminiscent of various ages from the Miocene until the Plio-Pleistocene [16].

**Figure 1:**
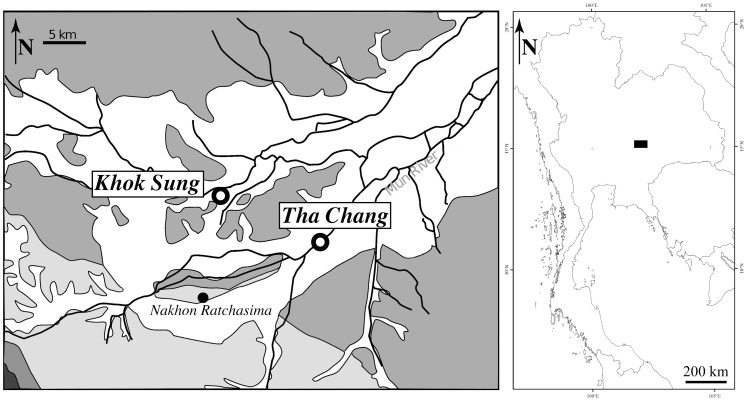
Map of northeastern Thailand showing the location of Khok Sung (modified from [**12**]).

In Thailand, paleontological excavations have been conducted by the Department of Mineral Resources and inhabitants of the Khok Sung municipality, Muang District, Nakhon Ratchasima Province, northeastern Thailand (Fig. 1) and yielded an assemblage of plant remains [17], mammals, turtles and *Gavialis* [18]. The mammal assemblage has not been studied yet but contains *Stegodon*, hyena, bovid and deer indicative of an Early Pleistocene age [18] as well as large silurids (JC pers. obs) and a large snake vertebra (JC and JEM, pers. obs.). The sedimentology of the Khok Sung locality was briefly described and figured in [18] and is composed of recent alluvial deposits of silts, sands, and gravels. The vertebrate-rich layer is situated about 8 m below the surface and is considered to be part of the PaleoMun river system. The locality is currently flooded and no longer accessible.

The excellent preservation of the *Gavialis* remains from the Khok Sung quarry represents an opportunity to precisely describe and appraise for the first time the affinities of this taxon in Thailand. The genus *Gavialis* comprises a single living species, *G. gangeticus* Gmelin 1789 [19], which today is restricted to the riverbanks of Bangladesh, India, Nepal and West Pakistan. The oldest fossil material allegedly referable to this genus has been recovered from the Miocene of Pakistan [20]. *Gavialis* is abundant in continental Pleistocene deposits of the Indo-Pakistani region, where several species have been erected but await revision. The genus was also reported from the Pleistocene of Java [13], and although very similar to the extant *G. gangeticus*, a recent study [15] has confirmed the attribution of the material from Java to a separate species, *G. bengawanicus* Dubois 1908 [13]. Despite the relatively recent existence of *Gavialis* in Myanmar, the fossil record of the genus appears scarce between Indo-Pakistan and Java [21]: the only known reports of the genus in this region to date are those of [4] and [5], but the age of these fossils is uncertain. Here, we fill this gap and describe exquisitely-preserved skull and mandibular remains from the Early Pleistocene of Khok Sung, Nakhon Ratchasima Province, Thailand. A close relationship with *G. bengawanicus* from Java is proposed on the basis of the sutural configuration of the rostrum and palate, alveolar count and the shape of the skull table. Despite the restriction of *Gavialis* to freshwater systems, recent proposals on the biogeography of the genus have put forward the possibility of marine dispersal [15], which is also postulated for other extinct gavialoids such as gryposuchines [22]. Nevertheless, we stress that a freshwater dispersal between the various regions involved remains a possible option, especially in light of Plio-Pleistocene drainage evolution in the eastern Himalayan syntaxis. Finally, sea-level drops can explain dispersal to Java before or during the Early Pleistocene across the shallow Sunda shelf.

## Methods

### Ethics statement

No live animals were used in this study. No specific permits were required for the described field studies. The Department of Mineral Resources, Bangkok, Thailand (DMR) regulates fossil collecting. In this study, specimens described were not collected by the authors. We studied specimens that are registered by the DMR and curated at Khok Sung subdistrict municipality.

### Institutional Abbreviations

DMR-KS, Khok Sung Collection, Department of Mineral Resources, Bangkok, Thailand; CD, ‘Collectie Dubois’, Naturalis, Nationaal Natuurhistorisch Museum, Leiden, Netherlands; RMNH, Naturalis, Nationaal Natuurhistorisch Museum, Leiden, Netherlands.

## Results

### Systematic paleontology

**Order Crocodilia** Gmelin 1789 [19]

**Superfamily Gavialoidea** Hay 1930 [23]

***Gavialis*** Oppel 1811 [24]

#### Emended diagnosis

The following diagnosis is based on skeletal characters only. For other characteristics, notably based on color or scalation, refer to [25], [26], [27]. The following character combination can be regarded as autapomorphic for the genus: longirostrine animal with a long flattened tubular rostrum (rostrum length representing between 70% and 75% of total skull length) containing well-separated alveoli (intra-alveolar space at least 1.5 times larger than adjacent alveolar diameter); alveolar count is 5 per premaxilla, 20–24 per maxilla and 21–26 per dentary; absence of disparity in alveolar size, dentition homodont and procumbent rostrally; upturned orbital margins; extremely wide interorbital region with frontal width largely surpassing rostral width just anterior to the orbits (or posterior from the distal end of the lacrimals); short and thin frontal process separating posterior region of nasals; frontal reaching but not entering in between the supratemporal fenestrae; premaxillae tapering posteriorly and excluding nasals from nares; lightly built skull table with extensive supratemporal fenestrae covering more than 15% of skull table surface area; bifid ventral portion of basioccipital, modified into a pair of massive tubera; pterygoid bullae; vertical and triangular infratemporal fenestrae hosting a quadratojugal spine on its posterior corner; short quadrate condyles, not extending posteriorly beyond the posterior margin of the skull table; small external mandibular fenestrae, representing about 10% of the mandibular length; spatulate anterior dentary; midline dorsal shield possessing six osteoderms per row; dorsal osteoderms with few large ovoid pits on dorsal surface; humeral and femoral shafts slender.

***Gavialis*** **cf.** ***bengawanicus*** Dubois 1908 [13]

#### Referred material

DMR-KS-201202-1, a complete skull and associated mandibles; DMR-KS-03-25-23, a skull missing the rostrum and much of the palate; DMR-KS-05-06-22-1, a pair of dentaries; DMR-KS-05-03-08-37, a jaw fragment with two teeth; DMR-KS-05-03-27-7, a right humerus; DMR-KS-05-03-26-7, DMR-KS-03-27-22, a right proximal portion of humerus; DMR-KS-05-03-27-6, a left distal portion of humerus; a left femur; DMR-KS-05-03-26-12, 05-03-26-40, 05-03-26-41; 05-03-27-23, 05-03-27-24, 05-03-27-25, six osteoderms; DMR-KS-12-03-05, three osteoderms.

#### Occurrence

Early Pleistocene of Thailand (*Stegodon* fauna), Khok Sung, Nakhon Ratchasima Province, northeastern Thailand (Fig. 1).

#### Diagnosis

See [15] for the diagnosis of the species *Gavialis bengawanicus*.

### Description

#### General description

**Figure 2:**
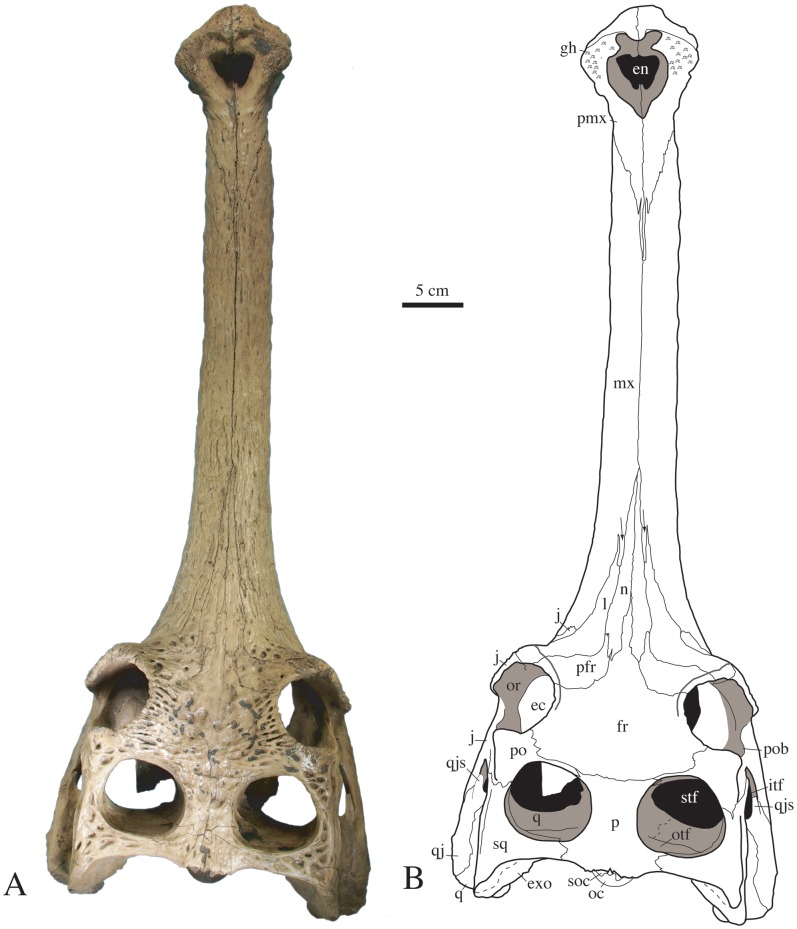
Skull of *Gavialis* cf. *bengawanicus* (DMR-KS-201202-1), from the Early Pleistocene of Khok Sung (Nakhon Ratchasima Province, Thailand), in dorsal view. Abbreviations: ec, ectopterygoid; en, external nares; exo, exoccipital; fr, frontal; gh, ghara fossa; itf, lower temporal fenestra; j, jugal; l, lacrimal; mx, maxilla; n, nasal; oc, occipital condyle; or, orbit; otf, orbitotemporal foramen; p, parietal; pfr, prefrontal; po, postorbital; pob, postorbital bar; pmx, premaxilla; q, quadrate; qj, quadratojugal; qjs, quadratojugal spine; soc, supraoccipital; stf, supratemporal fenestra; sq, squamosal.

**Figure 3:**
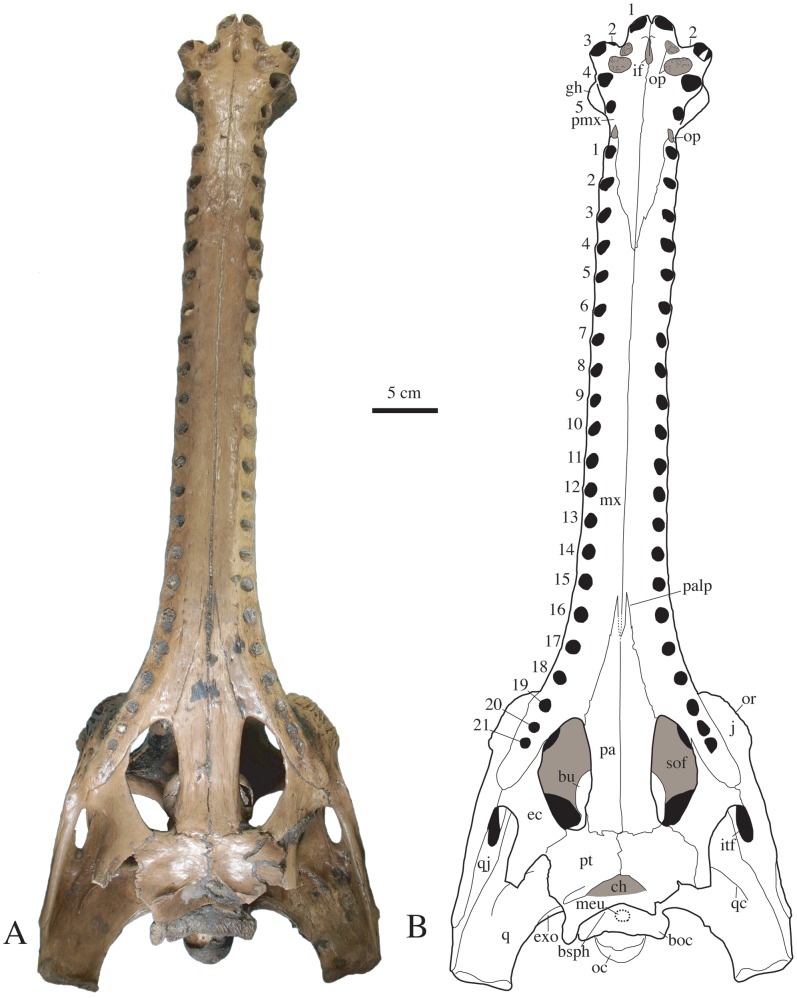
Skull of *Gavialis* cf. *bengawanicus* (DMR-KS-201202-1), from the Early Pleistocene of Khok Sung (Nakhon Ratchasima Province, Thailand), in ventral view. Abbreviations: boc, basioccipital; bsph, basisphenoid; bu, pterygoid bulla; ch, choanae; ec, ectopterygoid; exo, exoccipital; gh, ghara; if, incisive foramen; itf, lower temporal fenestra; j, jugal; meu, median Eustachian opening; mx, maxilla; oc, occipital condyle; op, occlusal pit; or, orbit; pa, palatine; palp, palatine process; pmx, premaxilla; pt, pterygoid; q, quadrate; qc, quadrate crest; qj, quadratojugal; sof, suborbital fenestra; sq, squamosal.

**Figure 4:**
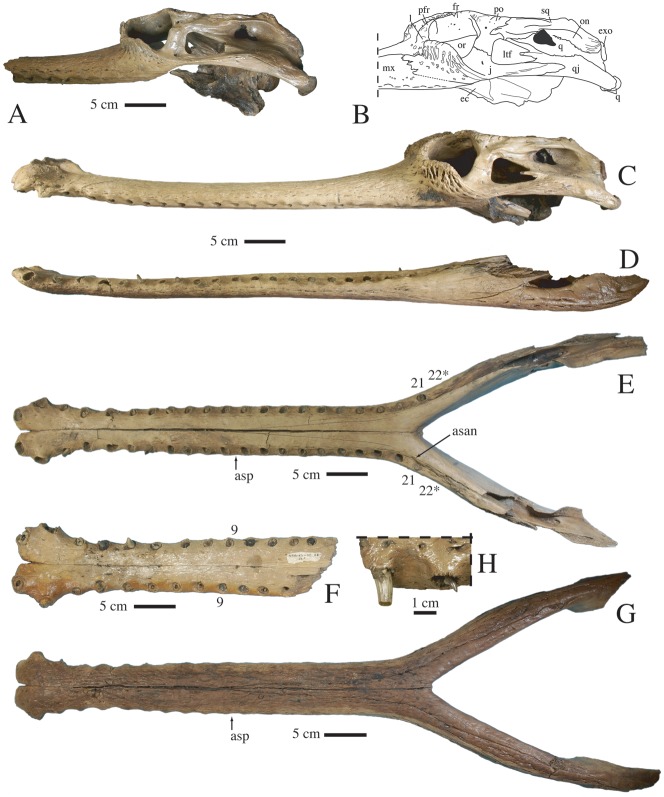
Skull and mandible of *Gavialis* cf. *bengawanicus* from the Early Pleistocene of Khok Sung (Nakhon Ratchasima Province, Thailand). A, right lateral view (mirrored for comparison) of DMR-KS-03-25-23. B, line drawing from the left lateral view of the posterior portion of the skull of DMR-KS-201202-1. C, left lateral view of skull of DMR-KS-201202-1. D, E, G, mandible of DMR-KS-201202-1 left lateral (D), occlusal (E), and ventral (G) views. F, incomplete dentaries (DMR-KS-05-06-22-1) in occlusal view; H, detail of the dentition in lateral view as seen on the maxillary fragment DMR-KS-05-03-08-37. Abbreviations: asan, anterior tip of surangular; asp, anterior tip of splenial; ec, ectopterygoid; exo, exoccipital; fr, frontal; j, jugal; l, lacrimal; ltf, lower temporal fenestra; mx, maxilla; on, otic notch; or, orbit; pfr, prefrontal; po, postorbital; q, quadrate; qj, quadratojugal; sq, squamosal.

**Figure 5:**
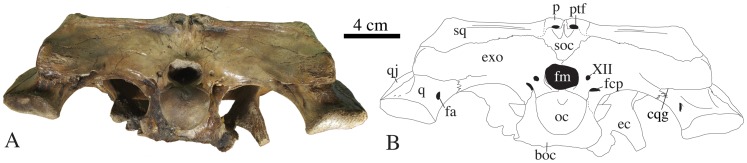
Skull of *Gavialis* cf. *bengawanicus* (DMR-KS201202-1) from the Early Pleistocene of Khok Sung (Nakhon Ratchasima Province, Thailand) in occipital view. Abbreviations: boc, basioccipital; cqg, cranioquadrate groove; ec, ectopterygoid; exo, exoccipital; fa, foramen aëreum; fcp, foramen caroticum posterius; fm, foramen magnum; oc, occipital condyle; p, parietal; ptf, post-temporal foramen; q, quadrate; qj, quadratojugal; soc, supraoccipital; sq, squamosal; XII, foramen for cranial nerve XII.

The two skulls from Khok Sung offer fine details of their anatomy. Ornamentation is light on the rostrum and anterior to the orbits, consisting of shallow furrows. It is also well expressed in the interorbital region and on the postorbital and squamosal, where it consists of large and deep circular to furrow-like grooves. The jugal bears a few sparse pits, but ornamentation is absent from the quadratojugal. Smaller pits are present on the dorsal surface of the premaxilla. The orbital rim is ornamented with deep furrows radiating from the orbital margin. The rostrum of the complete specimen (DMR-KS-201202-1, Figs. 2, 3, 4) is long, tubular, slender and its ventral surface is convex in lateral view. In this specimen, the external nares are surrounded by a large fossa for the insertion of a soft-tissue protuberance, or “ghara”, indicating that this specimen is a male. Skull proportions are close to those of *G. gangeticus* and *G. bengawanicus* (see Table 1 for measurements). The orbital and skull table regions are characteristic of the genus *Gavialis* in becoming suddenly wider by comparison to the rostrum. The orbits protrude dorsolaterally from the skull, their lateral margins consisting of a thick lamina formed by the prefrontal, lacrimal and jugal. The interorbital space is wider than the diameter of one orbit in dorsal view, and is also wider than the space between the supratemporal fenestrae. The supratemporal fenestrae are large and subcircular. In occipital view, the dorsal surface of the skull table is flat (Fig. 5). The infratemporal fenestra is triangular in shape and faces almost entirely laterally.

**Table 1:** Skull and mandibular dimensions (in cm) of *Gavialis* cf. *bengawanicus* from the Early Pleistocene of Khok Sung (Nakhon Ratchasima Province, Thailand).

	DMR-KS-03-25-23	DMR-KS-201202-1
Length of skull, from tip of snout to basioccipital condyle	?	72
Maximal width of skull, across quadrates	27	25.3
Length of snout, from anterior border of orbits to anterior border of premaxilla	?	53
Length of post-snout region, from anterior border of orbit to posterior edge of cranial table	19	18
Maximal width of snout at tip of nasals	6.9	6.2
Maximal length of naris	?	2.6
Maximal width of naris	?	3.7
Diameter of orbit parallel to orbital rim	5.8	5.7
Width between medial hemicondyles	17	16.6
Interorbital width	9.2	9.7
Length of cranial table, in between supratemporal fenestrae	10.5	9.9
Width of cranial table, across centers of supratemporal fenestrae	19.2	19.2
Maximal length of supratemporal fenestra	6.8	5.6
Maximal width of supratemporal fenestra	6.9	6.5
Interfenestral width	2.9	3.7
Length of ventral border of infratemporal fenestra	4.6	4
Length of incisive foramen	?	1.6
Width of incisive foramen	?	0.7
Length of long axis of suborbital fenestra	?	8
Length of short axis of suborbital fenestra	?	3.4
Interfenestral width of palatines	?	5
Width of choanae	?	4.5
Width across basioccipital ventral surface	7.4	6.9
Ghara width	?	9.5
Occipital condyle width	4.5	3.8

#### Skull

**Figure 6:**
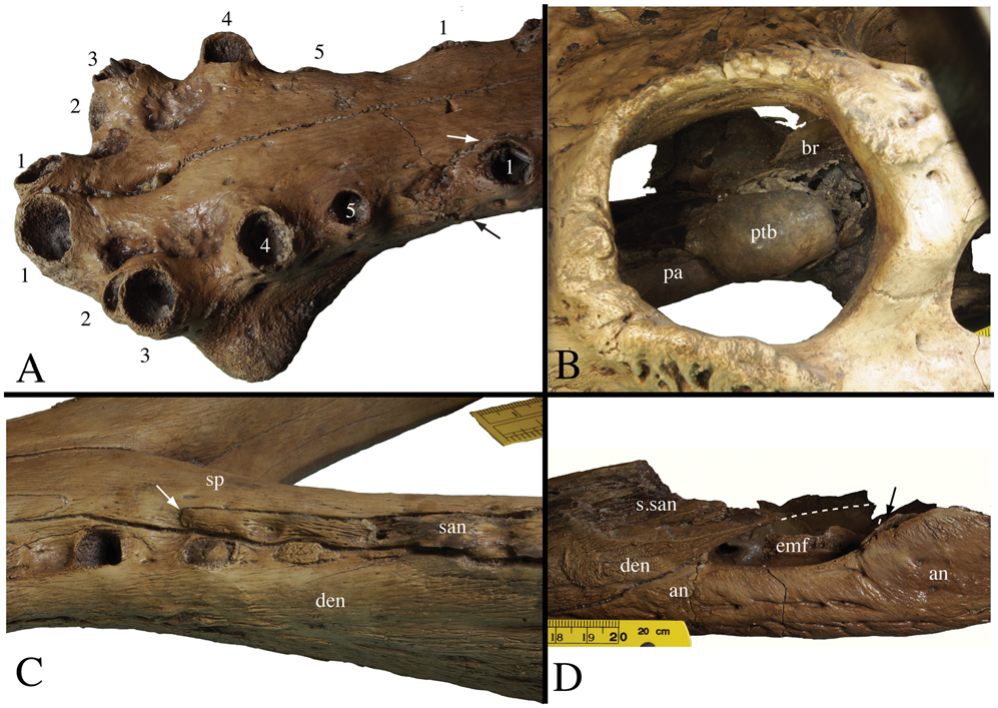
Skull and mandibular details of *Gavialis* cf. *bengawanicus* (DMR-KS-201202-1) from the Early Pleistocene of Khok Sung (Nakhon Ratchasima Province, Thailand). A, premaxillary toothrow in left oblique view. The arrows follow the premaxillary-maxillary suture. B, left lateral view of the pterygoid bullae through the orbit. C, lateral oblique view of the left posterior mandibular tooth row. The arrow points to the anterior process of the surangular. D, left lateral view of the posterior portion of the mandible. The arrow points to the descending process of the surangular along the posterior corner of the external mandibular fenestra. Abbreviations: an, angular; br, basisphenoid rostrum; den, dentary; emf, external mandibular fenestra; pa, palatine; ptb, pterygoid bulla; san, surangular; sp, splenial; s.san, suture for surangular.

The premaxilla (complete in DMR-KS-201202-1) is laterally expanded in comparison to the rest of the rostrum (Fig. 2). This shape is mostly due to the development of a depression accompanied by a roughened area around the margin of the external nares, which overhangs the ventral portions of the premaxillae. This perinarial depression is particularly well developed, being wider than long. The external nares are heart-shaped. The anterior margin of the perinarial depression presents a pair of shallow depressions. By contrast, the lateralmost tip of the perinarial depression is not smooth but presents instead a rough porous bone surface most probably serving as an anchor for the musculature of the narial excrescence or ghara [28]. The perinarial depression is also heart-shaped and discontinuous on the anterior sutural area. The premaxillae encompass the entire margin of the external nares and form the anterior part of the tubular snout. The premaxillae taper to a point posterior to the level of the fifth maxillary alveolus. Each premaxilla possesses five alveoli. The collars of the first four alveoli are almost tubular. In the first and second alveoli, the collar is oriented anteroventrally, but is oriented lateroventrally in subsequent alveoli. The second alveolus is small (Fig. 6A) and is contiguous with the larger third alveolus. As a result, the second alveolus is not visible in ventral view. The fifth alveolus is in line with the maxillary tooth row. Posterior to the fifth alveolus, the premaxilla possesses a posterior process that remains wide until the level of the third maxillary alveolus. From this point, the process tapers medially and the posterior extensions of the premaxillae terminate at the level of the fourth maxillary alveolus. The incisive foramen consists of a narrow incision opening at the level of the second premaxillary alveolus. Its anterior margin presents two peg-like bony outgrowths that meet medially. Two pairs of occlusal pits are present on the ventral margins of the premaxillae (Fig. 6A). The first is the smallest and deepest, and is located between the first and second alveoli. The second is about three times larger and much shallower, and occurs between the third and fourth alveoli. Several small reception pits resulting from the occlusion with the dentary dentition mark these occlusal pits, thus giving them an irregular surface.

**Figure 7:**
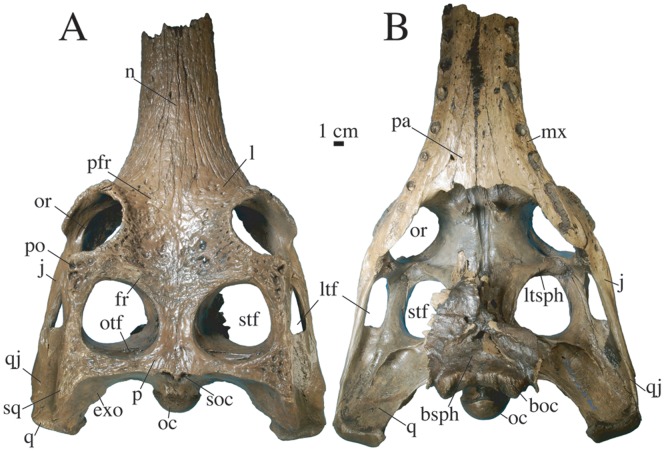
Right posterior maxillary tooth row of *Gavialis* cf. *bengawanicus* (DMR-KS-201202-1) from the Early Pleistocene of Khok Sung (Nakhon Ratchasima Province, Thailand), in occlusal view. Arrows indicate interalveolar occlusal pits.

The straight maxilla forms the majority of the tubular rostrum (Figs. 2, 3). In dorsal view, the maxillae meet medially along about half of the rostrum length due to the presence anteriorly of the premaxillae and posteriorly of the frontal. The maxilla has a long posterior process that separates the nasal from the long anterior tip of the lacrimal, as in *G. gangeticus*. Ventrally, the maxillae meet one another medially for a slightly longer length than on the dorsal surface, and have a convex surface. In lateral view, the premaxillomaxillary suture is vertical for a short distance and becomes oblique near the dorsal and ventral surfaces. This suture hosts two small and contiguous occlusal pits. Each maxilla possesses twenty-one alveoli of similar diameter, which are well separated from each other. All alveolar rims are slightly elevated and face slightly laterally. Shallow interalveolar pits are present in the posterior region of the tooth row, between the fifteenth and the sixteenth, the sixteenth and the seventeenth, and the seventeenth and the eighteenth alveoli (Fig. 7). The pits are not centered but are offset onto the anterior half of each interalveolar segment. The maxillae contribute broadly to the anterior margins of the suborbital fenestrae.

**Figure 8:**
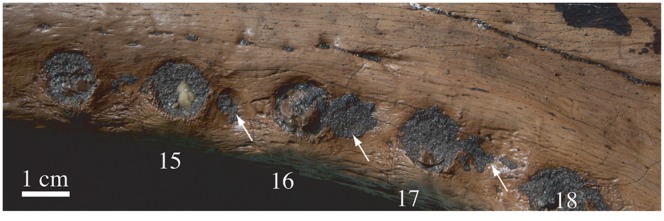
Partial skull of *Gavialis* cf. *bengawanicus* (DMR-KS-03-25-23) from the Early Pleistocene of Khok Sung (Nakhon Ratchasima Province, Thailand). Skull shown in dorsal (A) and ventral (B) views. Abbreviations: boc, basioccipital; bsph, basisphenoid; exo, exoccipital; ltf, lower temporal fenestra; j, jugal; l, lacrimal; ltsph, laterosphenoid; mx, maxilla; n, nasal; oc, occipital condyle; po, postorbital; or, orbit; otf, orbitotemporal foramen; pa, palatine; pfr, prefrontal; sq, squamosal; q, quadrate; qj, quadratojugal; sq, squamosal.

The nasal (Figures 2, 8B) is a short bone that reaches anteriorly as far as the level of the twelfth alveolus. It is narrow anteriorly and slightly wider in its posterior region. The nasals are deeply separated from each other by the anterior process of the frontal. Laterally, the nasal contacts the lacrimal along its posterior half and the maxilla in its anterior region. At its posterolateral end, the nasal contacts an elongate process of the prefrontal.

The long lacrimal possesses an anteriorly pointed process that extends within the maxillae (Fig. 2, 8B). It presents large rounded pits in its posterior region and protrudes along the anteriormost margin of the orbits. The lacrimal forms a broad contact with the maxilla in its anterolateral portion, contacts the jugal in the orbital region, and medially contacts the prefrontal and nasal.

The prefrontal (Figs. 2, 7B) is mostly ornamented with pits and furrows near the orbital margin. The prefrontal is very short in comparison with the lacrimal but possesses a thin anteromedial process that wedges between the nasal and lacrimal. The prefrontal is mediolaterally expanded and forms the anteromedial margin of the orbit.

The unpaired frontal (Figs. 2, 7B) consists of a wide and concave plate set between the orbits. Ornamentation consists of large and shallow pits in the posterior area and deep furrows near the orbital margin. The anterior triangular process of the frontal possesses a further thin and long process that extends between the nasals, reaching the level of the twelfth alveolus. The frontal is excluded from the anterior margin of the supratemporal fenestrae and forms the medial and posteromedial parts of the orbital rim.

The unpaired parietal (Fig. 2) forms the medial and posteromedial rims of the supratemporal fenestrae. It is ornamented with very shallow pits. Its dorsal surface is flat and does not overhang the fenestrae. Anteriorly, it broadly contacts the frontal just anterior to the margin of the supratemporal fenestrae. It also connects to a thin and long medial projection of the postorbitals. Posteriorly, the parietal does not expand much laterally and contacts the squamosal on the posterior temporal arch. The parietal delineates the posterior margin of the skull table with a slightly protruding outline.

The postorbital (Fig. 2) forms the anterior and anterolateral parts of the rim of the supratemporal fenestra. The ornamentation of the dorsal surface consists of a few large and deep pits. The anterior margin of the supratemporal fenestra consists of a thin projection of the postorbital, excluding the frontal from any contact with this fenestra. In dorsal view, the postorbital is short in comparison with the squamosal. The anterolateral margin of the postorbital is square in shape, the bone possessing a process that extends within the dorsal rim of the orbits (Figs. 2, 4B, C). The postorbital forms the anterodorsal margin of the infratemporal fenestra (Fig. 4B, C). Its descending process contacts at mid-length the jugal and forms the dorsal portion of the anteroposteriorly-expanded postorbital bar, which is smooth and set off from the skull table. A foramen opens laterally on the dorsal margin of the bar.

The squamosal contributes substantially to the temporal arch, thereby forming the lateral and posterolateral and most of the posterior parts of the margin of the supratemporal fenestra (Fig. 2). Anteriorly, the bone is slightly constricted and its dorsal surface lacks ornamentation. Several deep and elongate pits are visible on the posterodorsal corner of the bone. The posteriorly directed squamosal prong is short and descends onto the exoccipital (Fig. 4A, B, C). The squamosal forms the posterior and dorsal margin of the auditory meatus, where it contacts the quadrate (Fig. 4B, C). Anteriorly, the squamosal reaches the lateral surface of the postorbital bar. The squamosal is excluded from the dorsal margin of the infratemporal fenestra by the quadratojugal. The grooves for the ear-flap musculature are well pronounced just above the otic notch. Posteriorly, the otic notch is continuous with the external margin of the squamosal.

The jugal presents two distinct portions (Fig. 4B, C). Anteriorly, the jugal is deeply sculptured and forms the ventral laminar protrusion of the orbital rim. Posteriorly, the surface of the jugal is completely smooth and the jugal is inset from the orbital protrusion at the point where it contributes substantially to the postorbital bar. The jugal also extends as a flat rod along the ventral margin of the infratemporal fenestra. A foramen of varying size (small in DMR-KS-201202-1, large in DMR-KS-03-25-23) is present near the anteroventral corner of the infratemporal fenestra. The jugal extends over the lateral surface of the quadratojugal for a short distance.

The quadratojugal is a long bone that is constricted anterolaterally by the jugal and medially by the quadrate (Fig. 4B, C). The quadratojugal is completely devoid of ornamentation. It has a robust and long spine that participates in the dorsal margin as well as in the posterior corner of the infratemporal fenestra. In lateral view, the quadratojugal is dorsally arched and its posteriormost tip hides the quadrate but does not participate in the lateral hemicondyle.

The quadrate possesses a short ramus (Figs. 2, 3). In dorsal view, it does not extend far beyond the posterior margin of the skull table. Its ventral margin bears a faint crest close to the quadratojugal suture (Fig. 3). The primary head of the quadrate largely enters the supratemporal fenestra and floors the cranial aperture of the posttemporal canal. This foramen is dorsoventrally narrow and opens in the dorsal part of the vertical posterior wall of the supratemporal fenestra, where it occupies all the area. The quadrate approaches the most lateral tip of the laterosphenoid on the medial upper corner of the postorbital bar but does not contact it. The quadrate condyles are small. The foramen aëreum is small and located on the medial wall of the quadrate ramus (Fig. 4A). The quadrate seems to surround the dorsal, posterior and posteroventral margins of the anterolaterally facing foramen ovale. The extent of the prootic in forming the margin of the foramen ovale is unclear.

The laterosphenoid is best seen in DMR-KS-03-25-23 because the palate is missing (Fig. 8B). The laterosphenoids do not seem to meet medially as they are separated by a sulcus extending on the ventral margin of the frontal. The laterosphenoid is flat and square beneath the frontal and anteriorly reaches the prefrontal. Posteriorly, at the level of the postorbitals, it becomes dorsoventrally thick and expands laterally to reach the dorsomedial floor of the postorbital bar. The laterosphenoid also possesses a posterior process that extends along the medial surface of the lower margin of the supratemporal fossa, possibly taking part in the foramen ovale.

The pterygoid is almost complete in DMR-KS-201202-1, where its wings are missing (Fig. 3). The pterygoid-palatine suture lies at the posteriormost level of the suborbital fenestra. The pterygoid has a narrow contribution to the posteriormost corner of this fenestra. The choanae are fully enclosed within the pterygoids and sit well posterior to the palatine suture. They have a wide V-shaped aperture and open posteriorly, being floored by a laminar projection of the pterygoid. Just anterior to the choanae, the pterygoid presents a pair of depressions. The pterygoid seems to participate in the anteroventral portion of the foramen ovale. Its contact with the laterosphenoid is hidden by sediment but a contact seems to occur medially. The anterodorsal portion of the pterygoid has large bullae. The structure is hollow with thin walls and sits over the palatine bar. The bullae are visible through the suborbital fenestrae in ventral view (Fig. 6B).

The ectopterygoid forms most of the lateral margin of the suborbital fenestra (Fig. 3). It has a flat ventral surface, which connects anteriorly to the maxilla where it possesses a short thin process that extends along the medial maxillary margin to the level of the last alveolus. The ectopterygoid does not participate in the maxillary alveoli. Laterally, the ectopterygoid contacts the jugal and seems to possess a dorsal projection extending along the medial wall of the postorbital bar. The ectopterygoid contacts the pterygoid medially, but the nature of the contact along the pterygoid wing is unknown.

The palatine is a wide and flat beam forming the medial margin of the suborbital fenestra (Fig. 3). From the anterior margin of this fenestra, the palatine rapidly decreases in width and possesses a pointed process that extends within the maxilla to the level of the fifteenth to sixteenth maxillary alveoli. The palatinomaxillary suture is clearly W-shaped in DMR-KS-201202-1. Its geometry is incompletely known in DMR-KS-03-25-23 due to a ferruginous concretion hiding the suture.

The basisphenoid is visible in ventral view and its extent is relatively wide between the pterygoid and the basioccipital (Fig. 3). Its surface is still covered with sediment and the median Eustachian tube, which appears large and faces ventrally, is barely discernible. Anteriorly, the basisphenoid rostrum consists of a thin vertical lamina set in between the pterygoid bullae (Fig. 6B). In the most complete skull (DMR-KS-201202-1), the basisphenoid is difficult to discern on the lateral braincase wall due to the presence of sediment.

The basioccipital bears the occipital condyle, which has a diameter twice as large as the foramen magnum. Ventral to this condyle, the basioccipital plate is dorsoventrally short and, although incomplete, shows two pendulous and well-separated tubera (Fig. 4A).

The exoccipital forms most of the occipital surface of the skull (Fig. 4A). It is vertical and extends ventrally far along the basioccipital plate. Laterally, it possesses a lamina that extends over the squamosal. Ventrally, the exoccipital encloses the medially opened cranioquadrate groove as a result of a short lamina that extends over the quadrate. Cranial nerve XII opens on the exoccipital laterally to the foramen magnum.

The supraoccipital does not contribute to the dorsal surface of the skull table. It is widely exposed on the occipital surface of the skull but does not contact the foramen magnum (Fig. 4A). The posttemporal fenestra opens as a large slit at the junction between the parietal and the supraoccipital. Its ventral margin consists of a rugose supraoccipital process.

#### Mandible

The mandibles (DMR-KS-201202-1 and DMR-KS-05-06-22-1) show longitudinal furrows on the ventral surface. The external mandibular fenestra is as long as the infratemporal fenestra but is not dorsoventrally expanded. It is relatively small in comparison to the size of the mandible and its long axis is oblique and oriented anteroventrally (Fig. 4D–G).

The morphology of the dentary reflects that of the premaxilla and maxilla in being flat and tubular along most of its length and then spatulate in its anterior portion, comprising the first two alveoli (Fig. 4D, E). In cross section, the dorsal surface of the dentary is convex. The anteriormost region of the dentary is bifurcated in dorsal view (Fig. 4D, F). For most of its length, the dentary is perfectly straight. The first two alveoli are the largest in the tooth row. The first alveolus faces anterodorsally while all other alveoli face laterodorsally. The dentary contains 21 to possibly 22 alveoli, as suggested by the presence of a rugose bony infilling within the last alveolus (Fig. 6C). The symphysis extends to the level of the interval between the twentieth to twenty-first alveoli. Beyond this point, the tooth rows are V-shaped in dorsal view (Fig. 4E). Laterally, occlusal pits are visible in the posterior region of the tooth row, between the eighteenth and nineteenth, the nineteenth and twentieth, the twentieth and twenty-first, as well as between the twenty-first and twenty-second alveoli. These last two pits are partly on the surangular (Fig. 6C). The dentary possesses a posterior spiny projection extending between the angular and surangular, which contributes to the anterodorsal margin of the external mandibular fenestra (Fig. 6D). A second specimen, DMR-KS-05-06-22-1, shows that from the fifth alveolus onward, the right alveoli are not aligned with their counterparts on the left side (Fig. 4F).

The splenial participates deeply in the dentary symphysis, possessing a pointed process that extends anterior to the level of the twelfth alveolus. This contribution is visible both dorsally and ventrally (Fig. 4E, G). The splenial does not participate in the medial wall of the posterior tooth row, but completely hides the dentary in medial view of the bone. A large foramen opens at the end of the symphysis on the splenial suture. The splenial is also pierced by a small foramen that opens within the mandibular cavity. The ventral extension of the splenial is long, reaching beneath the angular.

The coronoid and most of the surangular are not preserved. The anteriormost process of the surangular is visible on the left ramus of DMR-KS201202-1, and projects along the medial wall of the last two alveoli. This surangular projection prevents the splenial from participating in the posterior tooth row (Fig. 6C). Sutural organization around the left external mandibular fenestra reveals that the surangular forms the dorsal margin. In addition, the surangular possesses a ventral process that excludes the angular from the posterior corner of the external mandibular fenestra (Fig. 6D).

The angular participates in the ventral margin of the external mandibular fenestra. Medially, it encloses the posterior intramandibular foramen. It also shows a well-developed laminar muscle scar at the bottom of the medial adductor fossa. The articular is not preserved.

#### Dentition

Two almost complete teeth are preserved in the third and seventh alveoli of the left dentary (DMR-KS201202-1). They are homodont and their morphology is best seen in two teeth preserved on a jaw fragment (DMR-KS-05-03-08-37, Fig. 4H). The teeth are pointed, deeply curved and bear a set of six to seven vertical ridges on each of their lingual and labial surfaces. The mesiodistal carinae are lingually deflected.

#### Postcranial skeleton

**Figure 9:**
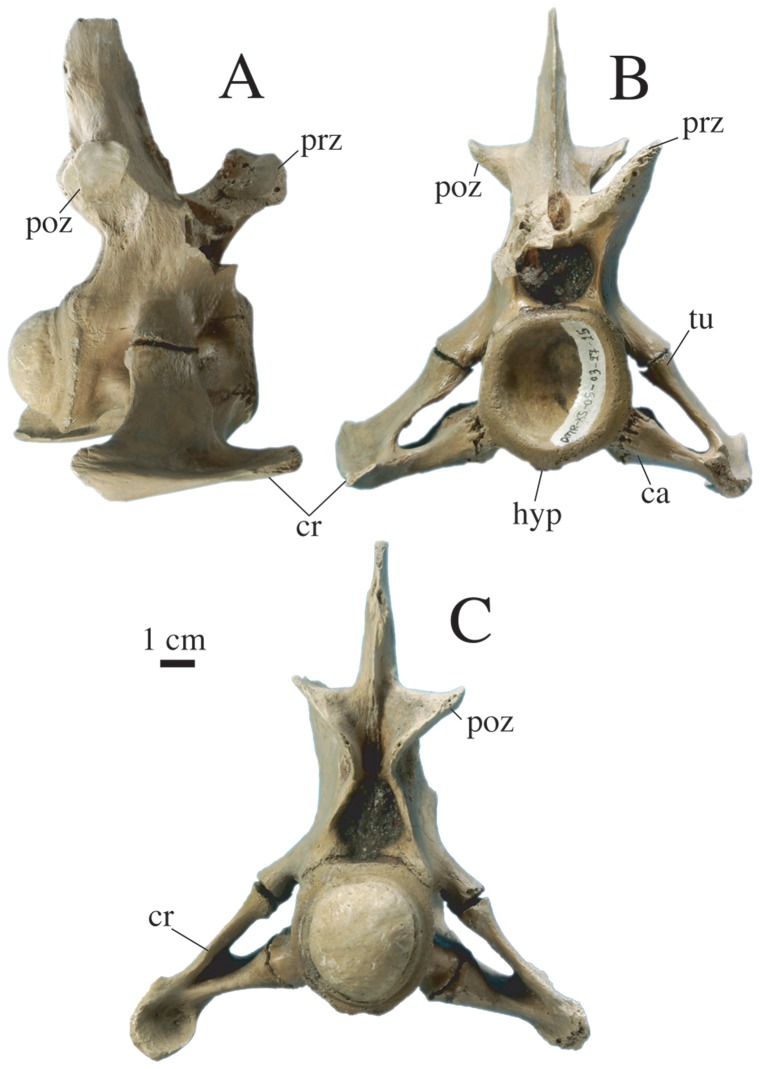
Cervical vertebra of *Gavialis* cf. *bengawanicus* (DMR-KS-05-03-27-15) from the Early Pleistocene of Khok Sung (Nakhon Ratchasima Province, Thailand). Vertebra shown in lateral (A), anterior (B) and posterior (C) views. Abbreviations: ca, capitulum; cr, cervical rib; hyp, hypapophysis; poz, postzygapophysis; prz, prezygapophysis; tu, tuberculum.

Two cervical vertebrae are preserved with cervical ribs attached (Fig. 9). The deeply procoelous centrum of DMR-KS-05-03-27-15 has a diameter of 4.2 cm. The ventral side of the centrum bears a hypapophyseal keel on its anterior portion. Diapophyses and parapophyses are well separated from each other and possess long pedicels that contact the tuberculum and capitulum of the cervical rib, respectively. The neural spine is slightly taller than the centrum and bears at its base the pre- and postzygapophyses, which are oriented at 45° to the coronal plane. The spinal process is mediolaterally compressed and anteroposteriorly expanded. Anteriorly, the neural canal is circular whereas it is triangular posteriorly. The cervical rib possesses an anterior process, projecting near the level of the anterior edge of the centrum. The height of the neural spine and the orientation of the articular surfaces of the zygapophyses indicate that this vertebra comes from the middle of the cervical series.

**Figure 10:**
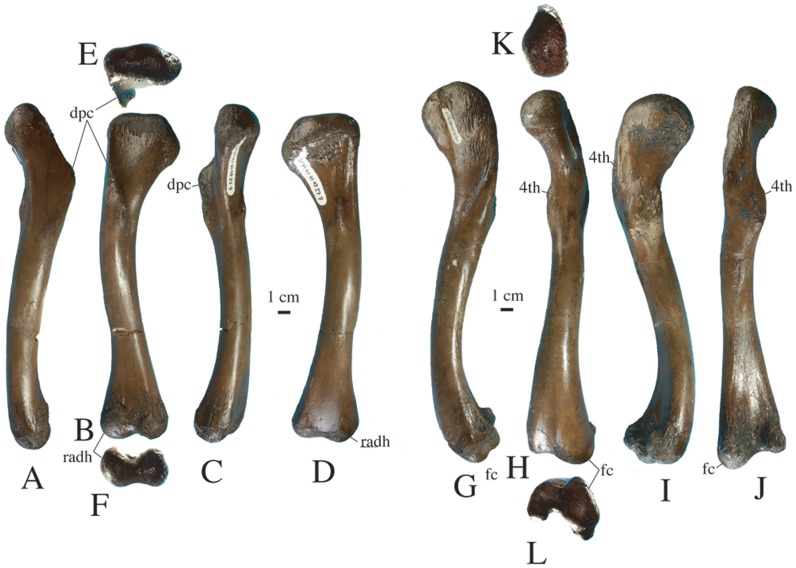
Limb elements of *Gavialis* cf. *bengawanicus* from the Early Pleistocene of Khok Sung (Nakhon Ratchasima Province, Thailand). Right humerus (DMR-KS-05-03-27-7) in lateral (A), anterior (B), medial (C), posterior (D), proximal (E), and distal (F) views. Left femur (DMR-KS-05-03-26-7) in lateral (G), anterior (H), medial (I), posterior (J), proximal (K) and distal (L) views. Abbreviations: 4th, fourth trochanter; dpc, deltopectoral crest; fc, fibular condyle; radh, radial hemicondyle.

Three humeri are preserved, but only a right one is complete (DMR-KS-05-03-27-7, Fig. 10). This humerus is 25.8 cm long and has a mid-shaft diameter of 2.9 cm. In anterior view, the humerus is curved along its medial margin in the proximal part of the shaft. The lateral edge of the bone is slightly convex but becomes strongly concave distally. The deltopectoral crest is well developed on the anterolateral edge, close to the proximal head of the humerus. The radial hemicondyle is about twice as large as the ulnar hemicondyle.

A complete left femur (DMR-KS-05-03-26-7, Fig. 10) is 30.1 cm long with a mid-shaft diameter of 3 cm. It is sigmoidal in lateral view. The greater trochanter is almost indistinct from the posterolateral margin. The fourth trochanter is well developed on the medial side of the shaft, near the posterior margin of the bone. It is located on the proximal portion of the shaft. The distal end of the femur is divided by a deep groove on its posterior surface, separating the much-enlarged fibular condyle from the tibial condyle. The fibular condyle bears a posterior protrusion.

**Figure 11:**
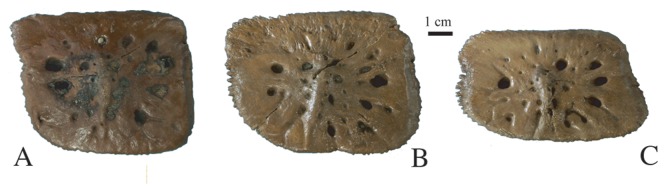
Dorsal osteoderms of *Gavialis* cf. *bengawanicus* from the Early Pleistocene of Khok Sung (Nakhon Ratchasima Province, Thailand), in dorsal views. A, DMR-KS-05-03-27-23. B, C, DMR-KS-12-03-05.

Thirteen dorsal osteoderms were found, but only nine have been accessioned and have specimen numbers. Some of the osteoderms are square and the majority of them are rectangular, being wider than long (Fig. 11) and flat to slightly convex. They are relatively thin and bear only a few relatively large pits on their dorsal surface. A short median keel is present on the dorsal surface, which does not spread anteriorly or posteriorly. The edges of these osteoderms are irregular, bearing small spiny outgrowths possibly indicating the presence of a suture. The ventral surface shows striae for epaxial muscle attachments as well as one or two relatively large foramina per osteoderm.

### Comparison and affinities

The fossil record of the genus *Gavialis* appears diverse in the Indo-Pakistani area with seven species described so far [20]. Four of these species are from the Miocene: *G. breviceps* Pilgrim 1912 [29], *G. pachyrhynchus* Lydekker 1886 [30], *G. curvirostris* Lydekker 1886 [30] and *G. browni* Mook 1932 [31]. Another two species are from the Mio-Pliocene: *G. hysudricus* Lydekker 1886 [30] and *G. lewisi* Lull 1944 [32]. Finally, *G. leptodus* Cautley and Falconer 1836 [33] is from the Plio-Pleistocene. A preliminary review of the literature and our description here of new remains from Thailand referable to the genus *Gavialis* led us to propose an emended diagnosis for the genus based on the work of Steel [26]. However, data for *G. browni* and *G. lewisi* were gathered from the literature only and first-hand observations would be desirable to pursue further work on this matter. The described material from Khok Sung, with its long tubular rostrum comprising 5 premaxillary alveoli and at least 20 maxillary alveoli, protruding orbital rims, wide interorbital space, large supratemporal fenestrae, pendulous basioccipital tubera and long mandibular symphysis incorporating at least 20 alveoli, can be assigned with confidence to the genus *Gavialis*. However, many taxa from the Indo-Pakistan area originally referred to *Gavialis* are either too incomplete for accurate taxonomic assignment or do not display the characteristics of the genus. Further work is required to assess this proposition and clarify synonymy issues (see also [15]), but according to the diagnosis proposed here, some of these species may not be included in the genus *Gavialis*.

*G. leptodus* is too fragmentary to allow a reliable identification, being represented by a fragmentary mandibular rostrum (plate XXXII figure 2 in [30]; plate XXIX fig. 4 in [34]). Each premaxilla of *G. pachyrhynchus* hosts only three alveoli, a feature contrasting with that of *Gavialis* where the premaxilla hosts five alveoli. The morphology of the premaxilla of *G. pachyrhynchus* alone may be sufficient to exclude it from the genus *Gavialis*. Two other taxa, *G. breviceps* and *G. curvirostris*, are represented by relatively complete material. The premaxillae of *G. breviceps* are anteriorly damaged, but four alveoli are present and there is room for another one; thus, this count does not differ from that of *G. gangeticus* and *G.* cf. *bengawanicus*. On the other hand, the complete dentary of *G. breviceps* (plate XXIX figs 2, 3 in [29]) contains 20 alveoli, 15 of which are included in the mandibular symphysis. This is unlike the condition of *G. gangeticus* and *G. bengawanicus*, where the dentary hosts more alveoli (25–26 and 22–23, respectively) and where all but the last two alveoli are included into the mandibular symphysis. *G. curvirostris* is known by an incomplete dentary but a complete maxillary tooth row (plate XXVIII in [29]) is short, possessing 17 alveoli. The abbreviated tooth rows of *G. breviceps* and *G. curvirostris* are therefore not comparable to those of *G. gangeticus* and *G. bengawanicus*. This conclusion is also corroborated by the morphology of the interorbital space of *G. curvirostris*, which is narrow compared to that of *G. gangeticus* and *G. bengawanicus*. The preserved maxillary tooth row of *G. browni* lacks its anteriormost margin and the total preserved maxillary alveolar count is 16. Mook [31] reconstructed the maxillary tooth row as having a total of 18 alveoli. However, the total count is unknown and whether *G. browni* most resembles *G. breviceps* and *G. curvirostris* with respect to this particular feature cannot be assessed. Nevertheless, *G. browni* displays three alveoli adjacent to the suborbital fenestra. This condition is restricted to one or two alveoli in *G. gangeticus* and *G. bengawanicus*. *G. hysudricus* is known from a relatively complete maxilla containing 23 alveoli (plate XXXII, fig. 1 in [30]), a count similar to that of *G. gangeticus* and slightly higher than *G. bengawanicus*. Lydekker [30] also referred a posterior portion of a skull to *G. hysudricus*, but in the absence of overlapping areas, there is no morphological evidence for this assignment. *G. hysudricus* may be similar to *G. gangeticus* at least in tooth count, but if Lydekker's assignment of the posterior portion of skull to *G. hysudricus* is correct, this taxon is not closely related to *G. gangeticus*. In fact, Lydekker's posterior skull table is similar to *G. curvirostris* and *G. browni* in displaying a relatively narrow interorbital space by comparison with *G. gangeticus* and *G. bengawanicus*. Indeed, in *G. gangeticus* the orbits increasingly protrude [35] and the interorbital space widens during the course of ontogeny (JEM, pers. obs.), but all of the skulls figured for the above-cited species are large enough to discount a juvenile state. *G. hysudricus* may well be a composite taxon and the possibility that a yet unnamed taxon close to *G. gangeticus* might have been present in the Mio-Pliocene of the Siwalik Hills should be evaluated. To sum up, *G. leptodus* is too fragmentary to be compared, whereas *G. pachyrhynchus*, *G. breviceps*, *G. browni* and *G. curvirostris* depart from the morphology of *G. gangeticus* and *G. bengawanicus*, suggesting the need for a reassessment of whether they should be considered members of the genus *Gavialis*. Finally, *G. hysudricus* may represent a valid species of *Gavialis* but clearly requires revision. The Khok Sung gharial is therefore the first species that can be confidently compared to the two best known members of this genus: the extant species *G. gangeticus* and the extinct Pleistocene species *G. bengawanicus* recently revised by Delfino and De Vos [15]. Of particular interest is also *G. lewisi* Lull 1944 [32] from the Late Miocene–Early Pliocene of Siwaliks, which will be compared below based on data from the literature [32], [36].

Because *G. gangeticus*, *G. bengawanicus* and the Khok Sung gharial are very similar in many respects, it is therefore necessary to analyze the variability of characters that are diagnostic of the two already known species to allow the correct taxonomic assignment of the specimens from Khok Sung. The postorbital protrusion and the width of the occipital condyle were used to distinguish *G. gangeticus* from *G. bengawanicus* [15] but the use of these characters is questionable: we find that a postorbital protrusion can be observed in all specimens of *G. gangeticus* and *G. bengawanicus*, as well as in the Khok Sung gharial. We are also unable to find any significant difference in the relative width of the occipital condyle between *G. gangeticus* and *G. bengawanicus*. Nevertheless, despite these similarities between *Gavialis gangeticus* and the form from Java, *G. bengawanicus* can definitely be considered as a distinct species.

The Khok Sung gharial is more similar to *G. bengawanicus* than to *G. gangeticus* on the basis of alveolar count, shape of the palatinomaxillary suture and shape of the skull table. Both the Khok Sung gharial and *G. bengawanicus* are characterized by a smaller number of maxillary alveoli (see Table 2) compared with *G. gangeticus*. This alveolar count has consequences for the relative positions of the premaxilla, splenial and symphysis with the tooth row, which are most similar between the Khok Sung gharial and *G. bengawanicus* (Table 2). This similarity is further corroborated by the shape of the palatinomaxillary suture, which is W-shaped in both the Khok Sung gharial and *G. bengawanicus* while it is V-shaped in *G. gangeticus* (fig. 2 in [15]). With regard to the skull table, the temporal arches sit ventrally to the intertemporal bar in *G. gangeticus* only. By contrast, the skull table is planar in both *G. bengawanicus* and the Khok Sung gharial. Comparison of the shape of the supratemporal fenestrae is also informative for DMR-KS-03-25-23 (Fig. 6A) because it presents the mature condition with the frontoparietal suture included in the anterior rim of the fenestrae (see [37]). Here, the outline of the supratemporal fenestrae is subcircular as in *G. bengawanicus* (CD9+1617a; fig. 1A, B in [15]). This is unlike the adult condition exhibited by *G. gangeticus* where the supratemporal fenestrae are slightly compressed anteroposteriorly and square-like in outline.

**Table 2:** Comparison of alveolar position (after [15]) and other characters between the extant *Gavialis gangeticus*, *Gavialis bengawanicus* from the Early Pleistocene of Java and *Gavialis* cf. *bengawanicus* from the Early Pleistocene of Khok Sung (Nakhon Ratchasima Province, Thailand).

Characteristics	*G. gangeticus*	*G. bengawanicus*	Khok Sung *Gavialis*
Premaxillary alveoli	5	?	5
Maxillary alveoli	23 or 24	20 or 21	21
Dentary alveoli	25 or 26	22 or 23	21 (22)*
Maxillary teeth adjacent to the posteroventral processes of premaxillae	3 or 4	3	4
Maxillary alveoli between the posteroventral tip of premaxillae and anterior tip of palatines	14	12 or 13	11
Maxillary alveoli posterior to anterior rim of suborbital fenestra	1 or 2	1	2
Dentary teeth included in the mandibular symphysis	23 or 24	20	20
Dentary alveoli preceding the anterior tip of splenials	14 or 15	12	11
Dentary alveoli involved in the splenial symphysis	9	8	9
Dentary teeth adjacent to the anterior dorsal process of surangular	4 or 5	2 or 3	1 (2)*
Shape of the maxillopalatine suture	V	W	W
Supratemporal fenestrae outline	Square-like	subcircular	subcircular
Skull table in occipital view	sloping	planar	planar
Anterior extension of the lacrimal	variable	long	long
Posteromedial processes of the maxilla	Broad separation	Minor separation	Broad separation
Occlusal maxillary pits	present	unknown	present
Anterior prefrontal process	long	short	long

* Behind the last dentary alveolus (on both rami), an ellipsoid area is rugose and may represent a filled alveolus. Therefore, the alveolar count might be higher and is indicated between brackets (see also Fig. 3).

However, the similarity between the Khok Sung gharial and *G. bengawanicus* is not clear-cut. Two characters observed in the Khok Sung gharial are also observed in some specimens of *G. gangeticus*, namely the anterior extension of the lacrimal and the development of a posteromedial process of the maxilla separating the nasal from the lacrimal. Nevertheless, the anterior extent of the lacrimal is subject to variation in *G. gangeticus* and is therefore of little diagnostic value. The lacrimal is long in all known specimens of *G. bengawanicus* from Java [15] and in both skulls from Khok Sung but varies in *G. gangeticus*. The anterior extent of the lacrimals is short in an unnumbered *G. gangeticus* specimen from the Naturhistorisches Museum Basel but is long in RMNH 39576 (Fig. 2 of [15]). Second, the maxilla possesses a diminutive and blunt process that separates the lacrimal from the nasals in *G. bengawanicus* from Java (Fig. 2A in [15]). This is not the case in the Khok Sung gharial where a maxillary process broadly separates the anterior tip of the lacrimal from the lateral margin of the nasal (Fig. 2, 7A), a case exactly similar to the condition in *G. gangeticus* (e.g. Fig. 2B in [15]). Further uncertainties in assigning the Khok Sung gharial to a previously described species relate to the shape of the prefrontal and the presence/absence of occlusal pits in the maxilla. According to Delfino and De Vos [15], the anterior process of the prefrontal is long in *G. bengawanicus*, but this does not appear to be the case based upon the figure presented by these authors (Fig. 1 in [15]). By contrast, the anterior process of the prefrontal is clearly elongated in the Khok Sung gharial. Delfino and De Vos [15] did not mention any occlusal pits in the maxillae of *G. bengawanicus*. Although these pits are shallow, they are present in the posteriormost region of the tooth row of the Khok Sung gharial.

These characters illustrate that the number of specimens from Java and Khok Sung is too small to allow a full assessment of character variability. Nevertheless, the Khok Sung gharial shares many similarities with *G. bengawanicus* (see Table 2), notably the alveolar count, positions of the sutures relative to the alveoli, planar skull table, and palatine-maxillary sutural shape. However, the Khok Sung gharial does not exactly fit the character description for *G. bengawanicus* for the anterior extent of the lacrimal and the posteromedial process of the maxilla. For these reasons, we refer the Khok Sung specimens to *Gavialis* cf. *bengawanicus*.

Clarifying differences and similarities between *G. gangeticus* and *G. bengawanicus* provides new data on the affinities of *G. lewisi*. Although *G. lewisi* has been coded in previous phylogenetic analyses (see [38] and others), Delfino and De Vos [15] noted that *G. lewisi* Lull 1944 [32] shows no detectable differences in character codings from *G. gangeticus*. However, coding characters for a phylogenetic analysis does not provide a comprehensive account of the morphological subtleties of a taxon. Here, we agree with Norell and Storrs [36] that *G. lewisi* should not be considered as *G. gangeticus* (contra [39]). Comparing *G. lewisi* to *G. bengawanicus* and *G. gangeticus* reveals that *G. lewisi* is more similar to *G. bengawanicus* than it is to *G. gangeticus*. *G. lewisi* shares at least three diagnostic characters with *G. bengawanicus* or *G.* cf. *bengawanicus*. First, and contra to Delfino and De Vos [15], the skull table of *G. lewisi* is similar to that of *G. bengawanicus* because it is planar from an occipital view (see Fig. 8 in [36]). Second, the outline of the supratemporal fenestrae of *G. lewisi* is subcircular (Fig. 7 in [36]) and, as described by [36], these fenestrae are smaller than in *G. gangeticus*. Finally, *G. lewisi* hosts a pair of depressions on the pterygoids, located in front of the choanae. Although the pterygoid wings are not preserved in *G. bengawanicus* from Java [15], we describe a pair of depressions on the pterygoids of *G.* cf. *bengawanicus* from Khok Sung (Fig. 2). Such depressions are absent in *G. gangeticus*. Whether *G. lewisi* can still be considered as a separate species from *Gavialis bengawanicus* is an open question and although this preliminary comparative account certainly remains incomplete, it suggests the presence of *G. bengawanicus* or a closely allied form in the Pliocene of the Siwaliks. In conclusion, three species are provisionally included in the genus *Gavialis*: *G. gangeticus*, *G. bengawanicus* and *G. lewisi*.

## Discussion

As shown by the present study, the genus *Gavialis* can now be considered to be represented by fossils from the Early Pleistocene of two regions in Southeast Asia, namely Java, with *Gavialis bengawanicus*, and Thailand, with *G.* cf. *bengawanicus*. In addition, fossil remains of a crocodilian closely resembling the living gharial were reported from the Irrawaddy Valley, Myanmar (Burma) [4], [5], and Burmese specimens of unknown age and exact provenance housed in the Petrified Wood Museum of Nakhon Ratchasima (Thailand) further confirm the presence of fossil *Gavialis* in this area (JEM, EB pers. obs). These finds are important because they are from a region that is geographically intermediate between the Thai and Javanese localities and those in India and Pakistan that have yielded abundant remains of Neogene gharials. However, at the moment too little is known about the gharial fossils from Myanmar to accurately assess their significance. The oldest known *Gavialis gangeticus* is from the Pliocene of the Indian subcontinent [20], [30], [38] and any discussion of the biogeographical history of *Gavialis* must therefore be based principally on the records from India and Pakistan in the west and from Thailand and Java in the east.

If the distribution of fossil and living species of *Gavialis* is to be understood in terms of dispersal, two main options may be considered: either dispersal took place exclusively via river systems, in agreement with the freshwater habitat of the living *Gavialis gangeticus*, or marine dispersal along the coasts of the Indian Ocean also played a part. These alternative options are discussed below.

### Plio-Pleistocene distribution of *Gavialis*

The inferred origin of *Gavialis* in the Indo-Pakistan area appears reasonable when considering the Pliocene co-occurrence of *G. lewisi* [32] and *G. gangeticus* [30] in this region, although future discoveries from SE Asia will have to be taken into account. In addition, when revised, *G. hysudricus* may confirm this inference of an Indo-Pakistan origin. As demonstrated in the comparisons conducted herein, *G. lewisi* is closer to *G. bengawanicus* than it is to *G. gangeticus*. The possibility that *G. lewisi* is a junior synonym of *G. bengawanicus* should be evaluated but this close relationship indicates that close relatives of the Pleistocene *G. bengawanicus* from SE Asia occurred in the Siwalik area during the Pliocene. Because *G. bengawanicus* appears more similar to the Pliocene *G. lewisi* than to *G. gangeticus*, it may therefore be considered as having diverged from *G. lewisi* no later than the Early Pleistocene. From this viewpoint, a west to east dispersal of *Gavialis* can be proposed. Populations of *G. bengawanicus* in SE Asia could be regarded as a relict of a once larger population spread from Indo-Pakistan to Java. Faunal connections between adjacent drainage basins across Asia were stopped with the rise of the Himalayas (see below). After the Pleistocene, *G. gangeticus* would then have replaced *G. bengawanicus* in the Indo-Pakistan area only. Indeed, it cannot be excluded that populations of *G. gangeticus* and *G. bengawanicus* coexisted, even until recently. It is not impossible that genetic barriers between such populations may have not been complete, allowing gene flow to some extent, which could explain the close similarities of the Khok Sung gharial with both *G. gangeticu*s and *G. bengawanicus*. A survey of historical specimens from Myanmar would allow determination of whether *G. bengawanicus* still occurred there in the recent past.

*Gavialis* cf. *bengawanicus* was already present in mainland SE Asia in the Early Pleistocene (this study) and *Gavialis* sp. has been recovered in the nearby slightly older strata of Tha Chang. This is the oldest Thai record of the genus, which comes from an indeterminate interval of Plio-Pleistocene age [12], or possibly as old as the middle Miocene (see [16]), although this latter date appears too old given that the remains have been referred to *Gavialis* sp. As shown in the above comparison, the Miocene species from the Indo-Pakistan area referred to *Gavialis* have a narrow interorbital space. This is not the case of the material of *Gavialis* sp. from Tha Chang that most closely resembles the Plio-Pleistocene species of *Gavialis*. It is highly likely that most elements of the Tha Chang crocodilians are of Plio-Pleistocene age. *G. bengawanicus* occurs in the Early Pleistocene of Java and seems absent in the Late Pliocene–earliest Early Pleistocene, thus suggesting a possible arrival date in Java not earlier than the Early Pleistocene [15]. The Khok Sung locality is not precisely dated and it cannot be ascertained whether it is slightly younger or older than the *Gavialis*-yielding localities in Java.

Nevertheless, other occurrences of *Gavialis* from Nakhon Ratchasima Province [12] strongly suggest that, outside the Indo-Pakistan area, the genus was first present in Thailand, then in Indonesia. Assuming that the Southeast Asian representatives of *Gavialis* originated on the Indian subcontinent, marine dispersal to Java or Thailand involves in any case long distances, and there seems to be no special reason why Thailand should have been reached later or earlier than Java. Thus, a marine dispersal does not clarify the observed difference for the arrival of the taxon in either region.

### Explaining *Gavialis* dispersal through fluvial capture

**Figure 12:**
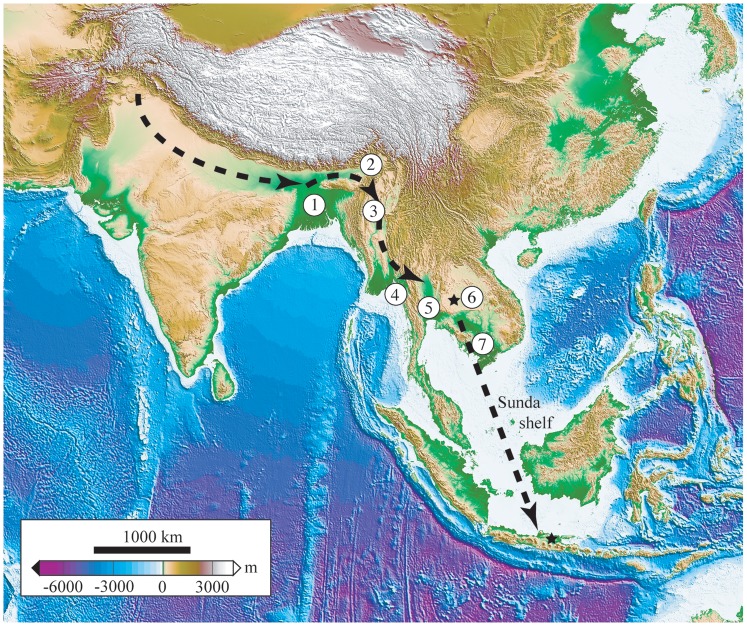
Hypothetic dispersal route of *Gavialis* spp. from their ancestral habitat in Indo-Pakistan toward SE Asia through the East Himalayan syntaxis. Definitive isolation of *Gavialis* population is represented by the mountain barriers separating the Salween and Chao Phraya basins and may have taken place during the latest Pliocene–earliest Pleistocene. 1, Ganges Delta; 2, Bhramapoutre Basin; 3, Irrawaddy Basin; 4, Salween Basin; 5, Chao Phraya Basin; 6, Chi and Mun rivers Basin; 7, Mekong Delta. Stars indicate the Early Pleistocene records of *Gavialis* in SE Asia (Khok Sung, Thailand and Java, Indonesia).

An alternative explanation for the dispersal of *Gavialis* from the Indian subcontinent to Thailand is fluvial capture (Fig. 12). Tectonic consequences of the Asia-India collision spread to SE Asia through a network of active faults, the most famous being those of the Red River complex. It is considered that much of today's high mountains of the eastern Himalayan range developed during the Pliocene and Pleistocene [40]. The main rivers (namely Tsangpo, Brahmaputra, Mekong, Salween, Irrawaddy, Chao Phraya and Yangtze) follow these faults and were diverted several times in the past by fault shearing as illustrated in Lacassin et al. [41]. It is therefore conceivable that connections and disconnections between fluvial systems occurred between the basins of the Ganges, Brahmaputra, Irrawaddy and Mekong as a result of this orogenic activity, as has been suggested by some authors [12], [40], [42]. On this basis, Attwood et al. [43] proposed a scenario to explain the dispersal of freshwater triculine gastropods and their parasites. They suggested a passage “into Thailand via the lower Irrawaddy and the extended Mekong-Salween River which flowed together during the Pleistocene (c. 1.5 mya)” (p. 15 in [43]). The same authors [43] also pointed out that triculine gastropods and their parasites occurred initially in India, indicating further connection between Thailand and the western provinces of India. The precise timing of hydrographic connections between the various basins is unknown, but taking into account that much fluvial reorganization took place during the Plio-Pleistocene, we view the occurrence of *Gavialis* in Thailand as possible evidence for faunal connection of aquatic habitats with the Irrawaddy and Ganges Basins through valleys that were formerly continuous but no longer exist. As for other taxa (gastropods and their parasites [43]; turtles [12]), we propose that *Gavialis* dispersed eastward, successively through the drainages of the Ganges, Brahmaputra, Irrawaddy, Mekong-Salween and finally Chao Phraya into Thailand before the Early Pleistocene, assuming that the dispersal occurred via fluvial capture rather than dispersal along the shoreline.

Nevertheless, the occurrence of *Gavialis* cf. *bengawanicus* in the Early Pleistocene of Khok Sung pertains to the Chi River system. Presently, Thailand possesses two main drainage basins. The first is located in the central part of the country and is known as the Chao Phraya Basin, which flows southward into the gulf of Thailand. The second is located on the northeastern Khorat plateau and includes the Mun and Chi rivers, reaching the Mekong at the Thai-Lao border. The Mun and Chi rivers are separated from the Chao Phraya Basin by the elevated western synclinal edge of the Khorat plateau, which prevents any possible connection between these two hydrographic basins. It was proposed that the Mun River flowed along the Thai-Cambodia border to the west in the past and probably reached the Chao Phraya river system, which was further connected to the Mekong, then flowing directly southward [12], [40], [43]. In that case, the occurrence of *Gavialis* in a tributary of the Chao Phraya River makes sense in regard to past hydrographic connections to the west with Myanmar and India.

Finally, *G. bengawanicus* may have reached Java during a low sea-level episode of the Early Pleistocene, which would be in agreement with the observation of Delfino and De Vos [15] that *G. bengawanicus* seems to be absent in the Late Pliocene–earliest Early Pleistocene of Java. In revising the data of Miller et al. [44], Woodruff and Turner [45] identified several episodes of sea-level fluctuations, with sea level ranging from −92 m to +10 m between 1 and 2 Ma. Dispersal of *G. bengawanicus* to Java is easily explainable by even minor sea level drops during the Early Pleistocene [45], sufficient to irrigate drainages on the Sunda shelf down to Java (Fig. 12). In the light of these values in sea level variation, the proposed emerged surface area would have been large enough to accommodate freshwater drainages and the arrival of *G. bengawanicus* in Java might also have taken place through fluvial dispersal.

### Physiological indications

The only living species of gharial, *G. gangeticus*, is a resident of freshwater habitats in Bangladesh, India, Nepal and Western Pakistan [21], [27], [46]. *G. gangeticus* demonstrates poor ability for land locomotion and crawls on sandy banks for basking and egg laying [21]. It is therefore considered the most aquatic of all living crocodilians. One physiological peculiarity of *G. gangeticus* is its very low secretory capacity for NaCl. This is unlike the genus *Crocodylus*, which has a demonstrated adaptability to the marine environment [47], [48]. The ability to cope with saltwater is considered plesiomorphic for Gavialoidea as suggested by the fossil record [22], [49] and by the presence of a keratinized tongue in *G. gangeticus* [48], [49]. *G. gangeticus* seems to have lost the ability to cope with saltwater, although admittedly its physiology has not been explored in detail [48]. There is one report of *G. gangeticus* in brackish water in India [50], but the same authors cast doubt on the validity of such an occurrence. Nevertheless, to our knowledge there are no reports of *Gavialis* venturing to sea naturally. If this does occur, it may be considered incidental. For example, the frequent floods during the monsoon season may result in gharials living in deltas being washed out to sea. Incidental delta hopping seems a possible explanation for short-distance dispersal of individuals along the coast between the Ganges delta and the Irrawaddy delta. By contrast, the known ecology of *Gavialis* seems inappropriate to explain long-distance dispersal from India to the deltas of SE Asia, which would involve prolonged marine dispersal (Fig. 12) to bypass mountain ranges separating the basins, and the distance to overcome by swimming would have been even greater during episodes of low sea level. Antagonistic behaviour of *Crocodylus* toward other crocodilian species might have limited the dispersal of *Gavialis* along the coastline, in areas in which *C. porosus* is already distributed. However, too little is known about such an interaction with modern populations of *Gavialis* to ascertain this possibility. A recent discussion on the capacity of *Gavialis* to cross marine barriers mentioned fossil records from Sulawesi and Woodlark (Papua New Guinea) that could not be explained without assuming a significant marine dispersal [15]. The attribution of these remains to *Gavialis* has not been fully demonstrated and the specimen from Woodlark was successively attributed to a new species of *Gavialis* (*Gavialis papuensis* De Vis 1905: [51]), to *Ikanogavialis* ([52], [53]; i.e. a gavialoid), to a malformed *Gavialis* [53], and to an *Euthecodon*-like crocodylid [54]. According to the published figures of the specimen (plate I in [52]), a long mandibular symphysis is the sole character in common with *Gavialis*, and is more generally shared with longirostrine taxa. The protruding alveoli are much reminiscent of those of *Ikanogavialis*, as established by [52]. Furthermore, the two associated dorsal osteoderms (plate II in [52]) present numerous pits and a straight anterior margin and depart from the morphology observed in *Gavialis*, which displays a concave anterior edge in some cases as well as a few large ovoid pits (fig. 5 of [15]). Therefore, an attribution of the Woodlark specimen to *Gavialis* seems unlikely. If an attribution proves correct for the Sulawesi occurrence of *Gavialis*, which was recovered from a coastal environment, it could be explained by short-distance dispersal from a nearby-emerged land in view of the extent of the Sunda shelf during low sea level episodes in the Pleistocene ([45], Fig. 12). In view of the limited amount of evidence for a dispersal of *Gavialis* by sea, we consider physical barriers, represented by mountain ranges and salt-water, as primary constraints on the distribution of the genus across Asia. As stated above, it cannot be excluded that *Gavialis* dispersed by sea for short distances, but dispersal through freshwater systems seems more likely.

## Conclusions

We recognize the presence of *Gavialis* cf. *bengawanicus* in the Early Pleistocene Chi River system at Khok Sung, northeastern Thailand. Comparison of this material with other specimens referred to the genus *Gavialis* of Miocene to Pleistocene age leads us to currently recognize three valid species within the genus: *G. gangeticus*, *G. bengawanicus* and *G. lewisi*. These last two species appear to be closely related, but a revision of *G. lewisi* needs to be carried out. The previous distribution of the genus encompasses an extensive area from Indo-Pakistan to Java. However, the presence of *Gavialis* in SE Asia (Thailand and Java) is relatively recent compared to the reported occurrences from the Indian subcontinent. Rather than dispersal by sea, we propose a vicariant hypothesis through fluvial captures of the eastern Himalayan syntaxis to explain the arrival of *Gavialis* in SE Asia. A separation of drainages by mountain uplift before the earliest Early Pleistocene may have caused the isolation of the *Gavialis* populations of Thailand from those of the Ganges-Irrawaddy basins. In light of this hypothesis, and taking into account the antiquity of both *G. gangeticus* and *G. lewisi*+*G. bengawanicus*, a survey of the fossil record of *Gavialis* in Myanmar will allow this idea to be refined, and further clarify the evolutionary history of the genus from India to Java.

## References

[1] Ducrocq S, Chaimanee Y, Suteethorn V, Jaeger J-J (1994) Age and paleoenvironment of Miocene mammalian faunas from Thailand. Palaeogeogr Palaeoclimatol Palaeoecol 108: 149–163.

[2] Ducrocq S, Chaimanee Y, Suteethorn V, Jaeger J-J (1995) Mammalian faunas and the ages of the continental Tertiary fossiliferous localities from Thailand. Journal Southeast Asian Earth Sci 12: 65–78.

[3] Tougard C, Montuire S (2006) Pleistocene paleoenvironmental reconstructions and mammalian evolution in South-East Asia: focus on fossil faunas from Thailand. Quaternary Sci Rev 25: 126–141.

[4] Buckland W (1828) Geological account of a series of animal and vegetable remains and of rocks, collected by J. Crawfurd, Esq. on a voyage up the Irawadi to Ava, in 1826 and 1827. Trans Geol Soc Lond 2: 377–392.

[5] Clift W (1828) On the fossil remains of two new species of Mastodon, and of other vertebrated animals, found on the left bank of the Irawadi. Trans Geol Soc Lond 2: 369–376.

[6] van der Maarel FH (1932) Contribution to the knowledge of the fossil mammalian fauna of Java. Weten Mededeelingen 15: 208.

[7] ter Haar C (1934) Toelichting bij Blad 58 (Boemiajoe) Geologische Kaart Van Java. Dienst Mijnbouw Nederlandsch-Indie. 65 p.

[8] von Koenigswald GHR (1935) Die fossilen Saugetierfauna Java. K Neder Akad Weten Amsterdam 38: 188–198.

[9] Bourret R (1941) Les tortues de l'Indochine. Notes de l'Institut Océanographique de l'Indochine. Stn Mar Cauda, Nhatrang, p. 235.

[10] Tong H, Claude J, Buffetaut E, Suteethorn V, Naksri W, et al.. (2006) Fossil turtles of Thailand, an updated review. In: Lü JC, Kobayashi Y, Huang GD, Lee Y-N, editors. Papers from the 2005 Heyuan International dinosaur symposium. Beijing: Geological Publishing House. pp. 183–194.

[11] Setiyabudi E (2009) An early Pleistocene giant tortoise (Reptilia; Testudines; Testudinidae) from the Bumiayu area, Central Java, Indonesia. J Fossil Res 42: 1–11.

[12] Claude J, Naksri W, Boonchai N, Buffetaut E, Duangkrayom J, et al (2011) Tertiary Reptiles of the Paleo-Mun River Valley in Northeastern Thailand. Ann Paléontol 3–4: 113–131.

[13] Dubois E (1908) Das Geologische alter der Kendeng-oder Trinil-fauna. Tijdschr K Neder Aard Gen, S 2 25: 1235–1270.

[14] Janensch W (1911) In:Selenka L, Blackenbraum M, editors. Die Reptilienreste (exkl. Schildkröten). Plate XII, XIII in Die Pithecanthropus-Schichten auf Java. Geologische und Paläontologische Ergenbisse der Trinil Expedition (1907 und 1908). Leipzig: Verlag von Wilhelm Engelmann. pp. 61–74.

[15] Delfino M, De Vos J (2010) A revision of the Dubois crocodylians, *Gavialis bengawanicus* and *Crocodylus ossifragus*, from the Pleistocene *Homo erectus* beds of Java. J Verteb Paleontol 30: 427–441.

[16] Hanta R, Ratanasthiena B, Kunimatsub Y, Saegusa CH, Nakaya DH, et al (2008) A new species of Bothriodontinae, *Merycopotamus thachangensis* (Cetartiodactyla, Anthracotheriidae) from the Late Miocene of Nakhon Ratchasima, North-eastern Thailand. J Verteb Paleontol 28: 1182–1188.

[17] Grote P (2007) Studies of fruits and seeds from the Pleistocene of northeastern Thailand. Cour For Sekenbg 258: 171–181.

[18] Chaimanee Y, Yamee C, Tian P, Khaowiset K (2005) Fossils and their managements at Ban Khok Sung, Muang District, Nakhon Ratchasima Province, NE Thailand. Academic report no. DMR 25/2005. Department of Mineral Resources, Bangkok: Thailand. 60 p. (In Thai)

[19] Gmelin JF (1789) Regnum animale. In: Beer GE, editor. Caroli a Linne Systema Naturae per Regna tri Naturae, Secundum Classes, Ordines, Genera, Species, cum Characteribus, Differentiis, Synonymis, Locis. Volume 1(3), Leipzig. pp. 1033–1516.

[20] Piras P, Kotsakis T (2005) A new gavialid from Early Miocene of south-eastern Pakistan (preliminary report). Rend Soc Paleontol It 2: 201–207.

[21] Whitaker R (2007) Members of the Gharial Multi-Task Force (2007) The Gharial: going extinct again. Iguana 14: 25–33.

[22] Buffetaut E (1982) Systématique, origine et évolution des gavialidés sud-américains. Géobios, mém sp 6: 127–140.

[23] Hay OP (1930) Second bibliography and catalogue of the fossil Vertebrata of North America. Carnegie Instit Washington Pub 390 2: 1–1074.

[24] Oppel M (1811) Die Ordnung, Familien und Gattungen der Reptilien als Prodrom einer Naturgeschichte derselben. Lindauer, Munich. 87 p.

[25] Wermuth H (1953) Systematik der rezenten Krokodile. Mitt Zool Mus Berlin 29: 375–514.

[26] Steel R (1973) Handbuch der Paläoherpetologie. Crocodylia. Teil 16. O. Kuhn (ed.). G. Fischer Verlag. Stuttgart.

[27] Trutnau L, Sommerlad R (2006) Crocodilians: their natural history and captive husbandry. Chimaira editions. 646 p.

[28] Martin BGH, Bellairs AD'A (1977) The narial excrescence and pterygoid bulla of the gharial, *Gavialis gangeticus* (Crocodilia). J Zool 182: 541–558.

[29] Pilgrim GE (1912) The vertebrate fauna of the Gaj series in the Bugti Hills and the Punhab. Memoirs of the Geological Survey of India. Palaeontol Indica N S 4: 1–83.

[30] Lydekker R (1886) Siwalik Crocodilia, Lacertilia and Ophidia. Palaeontol Indica 3: 209–240.

[31] Mook CC (1932) A new species of fossil gavial from the Siwalik beds. Am Mus Novit 514: 1–5.

[32] Lull RS (1944) Fossil gavials from North India. Am J Sci 242: 417–430.

[33] Falconer H, Cautley PT (1840) Notice on the remains of a fossil monkey from the Tertiary strata of the Siwalik Hills in the North of Hindoostan. Trans Geol Soc Lond 2: 499–504.

[34] Cautley PT, Falconer H (1836) The fossil gharial of the Siwalik Hills. Asiatic Res 19: 32–38.

[35] Piras P, Colangelo P, Adams DC, Buscalioni A, Cubo J, et al (2010) The *Gavialis-Tomistoma* debate: the contribution of skull ontogenetic allometry and growth trajectories to the study of crocodylian relationships. Evol Dev 12: 568–579. 21040423 10.1111/j.1525-142X.2010.00442.x

[36] Norell MA, Storrs GW (1989) Catalogue and review of the type fossil crocodilians in the Yale Peabody Museum. Postilla 203: 1–28.

[37] Vélez-Juarbe J, Brochu CA, Santos H (2007) A gharial from the Oligocene of Puerto Rico: transoceanic dispersal in the history of a non-marine reptile. Proc R Soc B 274: 1245–1254. 10.1098/rspb.2006.0455 PMC2176176 17341454

[38] Brochu CA (1997) Morphology, fossils, divergence timing, and the phylogenetic relationships of *Gavialis* . Sys Biol 46: 479–522. 10.1093/sysbio/46.3.479 11975331

[39] Langston W Jr (1965) Fossil crocodilians from Columbia and the Cenozoic history of the Crocodilia in South America. Univ Calif Pub Geol Sci 52: 1–169.

[40] Hutchison CS (l989) Geological Evolution of South East Asia. Oxford University Press, New York. 392 p.

[41] Lacassin R, Replumaz A, Hervé Leloup P (1998) Hairpin river loops and slip-sense inversion on southeast Asian strike-slip faults. Geology 26: 703–706.

[42] Brookfield ME (1998) The evolution of the great river systems of southern Asia during the Cenozoic India-Asia collision: Rivers draining southward. Geomorphology 22: 285–312.

[43] Attwood SW, Panasoponkul C, Upatham ES, Meng XH, Southgate VR (2002) *Schistosoma ovuncatum* n.sp. (Digenea: Schistosomatidae) from northwest Thailand and the historical biogeography of Southeast Asian *Schistosoma* Weinland, 1858. Sys Parasito 51: 1–19. 10.1023/a:1012988516995 11721191

[44] Miller KG, Kominz MA, Browning JV, Wright JD, Mountain GS, et al (2005) The Phanerozoic record of global sea-level change. Science 310: 1293–1298. 16311326 10.1126/science.1116412

[45] Woodruff DS, Turner LM (2009) The Indochinese-Sundaic zoogeographic transition: a description and analysis of terrestrial mammal species distributions. J Biogeogr 36: 803–821.

[46] Stevenson C, Whitaker R (2010) Indian Gharial *Gavialis gangeticus*. In: Manolis SC, Stevenson C, editors. Crocodiles. Status Survey and Conservation Action Plan. Third Edition. Crocodile Specialist Group: Darwin. pp. 139–143.

[47] Taplin LE, Grigg GC, Beard L (1985) Salt gland function in fresh water crocodiles: evidence for a marine phase in eusuchian evolution? In: Grigg G, Shine R, Ehmann H, editors. Biology of Australasian Frogs and Reptiles. R Soc New South Wales. pp. 403–410.

[48] Leslie AJ, Taplin LE (2001) Recent developments in osmoregulation of crocodilians. In: Grigg G, Seebacher F, Franklin CE, editors. Crocodilian Biology and Evolution. Chipping Norton, NSW: Surrey Beatty. pp. 265–279.

[49] Brochu CA (2003) Phylogenetic approaches toward crocodilian history. Annu Rev Earth Planet Sc 31: 357–397.

[50] Singh LAK, Bustard HR (1982) Geographical distribution of the gharial, *Gavialis gangeticus*, in Orissa, India. Brit J Herpetol 6: 259–260.

[51] De Vis CW (1905) Fossil vertebrates from New Guinea. Ann Queensland Mus 6: 26–31.

[52] Molnar RE (1982) A longirostrine crocodilian from Murua (Woodlark), Solomon Sea. Mem Queensland Mus 20: 675–685.

[53] Molnar RE (1993) Biogeography and phylogeny of the Crocodylia. In: Glasby CG, Ross GJB, Beesley PL, editors. Fauna of Australia: Volume 2a, Amphibia and Reptilia. Australian Government Publishing Service, Canberra. pp. 344–348.

[54] Rauhe M, Frey E, Pemberton DS, Rossmann T (1999) Fossil crocodilians from the late Miocene Baynunah Formation of the Emirate of Abu Dhabi, United Arab Emirates: osteology and palaeoecology. In: Whybrow PJ, Hill A, editors. Fossil Vertebrates of Arabia. New Haven, Connecticut: Yale University Press. pp. 163–185.

